# Is Vivisection Irreligious, Immoral, or Inhumane?

**Published:** 1892-06-25

**Authors:** 


*For Hospitals, Asylums, Infirmaries, Dispensaries, Medical Practitioners, Students, Nurses, and the Charitable Public.*

Vol. XII.—No. 300.] [June 25, 1892.

### Is Vivisection Irreligious, Immoral, or Inhumane?

At the annual meeting of the Anti-Vivisection Society, held recently, the Right Rev. Bishop Barry moved the following resolution: "That this meeting holds the practice of vivisection to be unwarrantable upon religious, moral, and humane grounds." Physiologists and medical men do not, and cannot, live in a world by themselves apart. They have to live with, to serve, and to be served by, their fellow-men. A general obligation rests upon them, as upon all intelligent beings, to recognise the kind of world they have been, without their will, born into, to properly appraise the conditions of their human environment; and to be as loyal as they can to good feeling and good fellowship.

Bishop Barry thinks that vivisection cannot be defended on religious, moral, and humane grounds. Our reply to the Bishop is that if vivisection cannot be defended on these grounds, it cannot be defended at all. For the one differentiating factor between human beings and other animals is that which consists in the trinity of religiousness, morality, and humanity. If Bishop Barry be right, vivisection is opposed to the very fundamental essence of human nature; and any practice whatsoever which is so opposed is bound to go down before the forward progress of civilised man, and ought to go down. In our judgment, vivisection can be defended on religious, moral, and humane grounds.

Is there any kinship between man and animals? And what is the nature of that kinship? The relationship is absolute and permanent; so much so that, except as an animal, man has never been known to live at all in this world, and under present conditions. Men and animals constitute one vast family, of which man is mentally at the head. This family lives by breathing the same air, walking on the same solid earth, eating the food that the earth produces, and drinking the water that flows in its rivers; men and animals are all warmed by the same sun, and canopied by the same sky. They have similar joys of fatherhood and motherhood, similar pains and grief in their daily life; and they share the pangs and agonies of death in common. It is evident that Nature, Providence, the Creator, or whatever name we choose to give the power that is interfused with our destinies, intended men and animals, at any rate up to the point of physical death, to share a common lot. That men and animals constitute one family is so obvious to every single-minded man, that it cannot for a moment be intelligently denied.

In civilised countries man has established something more than a mere blood and bone relationship with the lower animals; he has taken them under his fostering protection, at any rate, within certain limits. In the main the animals not only submit to this protection, they love it. How easily the high-mettled and swift-footed horse could unseat his rider and gallop off to the freedom of the plains and hills! Why does the dog, in almost all his varieties, choose the protection and companionship of man rather than the wild license and the fierce delights of the woods? All the domestic animals are joined together in a peculiar family relationship with man at their head. With him they share the vicissitudes of war and peace, of the summer's heat and the winter's cold, of family prosperity and adversity.

It will, doubtless, be said the "protection" of the lower animals by man is no protection at all, but only a selfish and interested guardianship for a limited time. That is, in a measure, most true. We keep animals alive and prosperous for our convenience; and we kill them, when the time comes, also for our convenience. Whilst they are alive, if we protect them and use them well, they have no ground of complaint against us if we kill them for legitimate purposes in the long run. If it be said that they have, then have we not ourselves precisely the same ground of complaint against the providence that rules over man, in that we ourselves are, one and all, ruthlessly called upon to yield up our lives at the appointed time, with no possibility of escape, or even of protest? We are as much under the power of the inevitable as the animals are; the only difference is that whilst they see their inevitable in us, we do not see our inevitable at all.

Is it the fact or is it not that a certain amount of sacrifice is demanded of all sentient creatures for the sake of one another? Is this sacrifice only and always of a voluntary kind? By no means! Nature makes her own exactions at the hands of the unwilling as well as of the willing. The unwilling outnumber the willing in the proportion of thousands to one, but the exactions are made without flinching for all that. Civilisation is more exacting than even nature, and family life the most exacting of all. On whose behalf do the average father and mother exercise most self-denial! Is it on behalf of the public, or of their own children? Let us come close to that Christianity of which Bishop Barry is so honoured a representative. Is it a religion in which sacrifice and self-sacrifice are unknown? Is it not the religion of all others in which sacrifice and self-sacrifice are carried to the sublimest heights? We are pointed to the Founder of Christianity as to one who, by sacrificing himself utterly and absolutely, both in life and in death, won the indefeasible right to ask all other noble intelligences to bring themselves up to the same heroic level of entire self-abnegation. So profoundly is Christianity a religion of sacrifice and self-sacrifice, that he who knows nothing of these things has never yet entered within the doors of even the elementary school of this wonderful faith.

But if animals are of one family with man, is it reasonable to suppose that they will share only a small part of his lot; only the part which is brighter and less responsible, and not the part that is sterner, but more noble and elevating? The whole history of man tends to show that the domesticated animals at any rate have had to share in their measure in the sacrifice and self-denial which have always been exacted from man. The ancient Jew, and other races of course, not only killed his animals for food, but slew them, without any sense of cruelty or incongruity, in order to offer them to his God. And his God permitted it. Did he not even command it?

The present writer has peculiar reasons for striving to defend vivisection on religious and moral grounds. He holds, as firmly as Bishop Barry can hold, that whatever cannot be defended on religious and moral grounds ought not to be defended at all. Moreover, he has seen much vivisection done; but he never saw any done cruelly; and, however this may be questioned, he feels bound to say that no teaching of any kind ever gave him so strong and profound a grip of physiology and of general biological science as his few months' experience in the vivisection room.

Bishop Barry and other cultured and reasoning men must surely be aware of one fundamental characteristic of their present cause. It is only the fringe of a cause which is a thousand times greater and more comprehensive than itself. That cause is nothing less than the complete emancipation of all animals from the control and protection of man. Has Bishop Barry ever lived for a number of years on a stock-breeding farm? Does he know by daily contact, as the present writer does, what goes on from week to week, and from month to month, among the young sheep, the cattle, the horses, the pigs, and even the fowls that run about the farm yard? Has he ever been to a coursing meeting, or seen the hounds kill a fox, or shot rabbits, or helped to butcher birds? All these things go together. In fixing upon the protection of animals from vivisection Bishop Barry is straining at the gnat and swallowing the camel. Is he prepared to be logical? Will he inscribe upon his banner this comprehensive motto: "The complete emancipation of all animals from the control and protection of man?"

The kind-hearted Bishop and his well-meaning friends are not the only people whose souls are oppressed with the manifold tragedies that the sun looks down upon every day on this earth of ours. We see the struggle, the life and death grapple that is of hourly occurrence. The biologist knows more of it than even the Bishop and the gentle ladies who support him. But can we alter the constitution of the world? Can we protest to the skies, and say to the Divine Ruler, "What doest Thou?" The Bishop must believe in the physiologist's honesty; in his intelligence and his humanity. He must leave the physiologist to stand or fall to his own master. It is wonderful how history repeats itself. But yesterday the religious man was asking for freedom to worship his God in his own way; to-day the physiologist has to ask the religionist for freedom to serve his brother man in his own way. "It is a strange world, my masters." "There are more things in heaven and earth than are dreamt of in your philosophy."

